# Preventing the Risk of Hospitalization for Respiratory Complications of Influenza among the Elderly: Is There a Better Influenza Vaccination Strategy? A Retrospective Population Study

**DOI:** 10.3390/vaccines8030344

**Published:** 2020-06-28

**Authors:** Silvia Cocchio, Tolinda Gallo, Stefania Del Zotto, Elena Clagnan, Andrea Iob, Patrizia Furlan, Marco Fonzo, Chiara Bertoncello, Vincenzo Baldo

**Affiliations:** 1 Department of Cardiac Thoracic Vascular Sciences and Public Health, University of Padua, Via Loredan 18, 35100 Padova, Italy; silvia.cocchio@unipd.it (S.C.); patrizia.furlan@unipd.it (P.F.); marco.fonzo@unipd.it (M.F.); chiara.bertoncello@unipd.it (C.B.); 2 Public Health Department, Udine Healthcare and University Integrated Trust, Via Chiusaforte 2, 33100 Udine, Italy; linda.gallo@asufc.sanita.fvg.it (T.G.); andrea.iob@asufc.sanita.fvg.it (A.I.); 3 Regione Friuli Venezi Giulia, Azienda Regionale di Coordinamento per la Salute, Via Pozzuolo 330, 33100 Udine, Italy; stefania.delzotto@arcs.sanita.fvg.it (S.D.Z.); elena.clagnan@arcs.sanita.fvg.it (E.C.)

**Keywords:** influenza, hospitalization, vaccination strategy, epidemiology

## Abstract

Influenza and its complications are an important public health concern, and vaccination remains the most effective prevention measure. However, the efficacy of vaccination depends on several variables, including the type of strategy adopted. The goal of this study was to assess the impact of different influenza vaccination strategies in preventing hospitalizations for influenza and its related respiratory complications. A retrospective cohort study was conducted on data routinely collected by the health services for six consecutive influenza seasons, considering the population aged 65 years or more at the time of their vaccination and living in northeastern Italy. Our analysis concerns 987,266 individuals vaccinated against influenza during the study period. The sample was a mean 78.0 ± 7.7 years old, and 5681 individuals (0.58%) were hospitalized for potentially influenza-related reasons. The hospitalization rate tended to increase over the years, not-significantly peaking in the 2016–2017 flu season (0.8%). Our main findings revealed that hospitalizations related to seasonal respiratory diseases were reduced as the use of the enhanced vaccine increased (*R*^2^ = 0.5234; *p* < 0.001). Multivariate analysis confirmed the significantly greater protective role of the enhanced vaccine over the conventional vaccination strategy, with adjusted Odds Ratio (adj OR) = 0.62 (95% CI: 0.59–0.66). A prior flu vaccination also had a protective role (adj OR: 0.752 (95% CI: 0.70–0.81)). Age, male sex, and H3N2 mismatch were directly associated with a higher risk of hospitalization for pneumonia. In the second part of our analysis, comparing MF59-adjuvanted trivalent inactivated vaccine (MF59-TIV) with conventional vaccines, we considered 479,397 individuals, of which 3176 (0.66%) were admitted to a hospital. The results show that using the former vaccine reduced the risk of hospitalization by 33% (adj OR: 0.67 (95% CI: 0.59–0.75)). This study contributes to the body of evidence of a greater efficacy of enhanced vaccines, and MF59-adjuvanted TIV in particular, over conventional vaccination strategies in the elderly.

## 1. Introduction

Influenza is a vaccine-preventable infectious disease that spreads all over the world, and the burden of this disease poses a serious public health problem for numerous countries. Seasonal influenza continues to cause a common acute respiratory disease, and 3–5 million cases of severe illness are estimated to occur every year around the world [1,2,3]. From 2010 to 2017, between 9.2 million and 35.6 million people in the United States became ill due to infection with the influenza virus, and between 140,000 and 710,000 required hospitalization [4]. In Europe, there were an estimated 17.8 million cases of influenza in 2018 [3].

In Italy, a sentinel surveillance system for influenza has been in operation since 2000. It is estimated that around 9% of the population is affected every season [5].

Flu-related disease can be complicated by several forms of pneumonia: primary viral pneumonia, secondary bacterial pneumonia, or combined viral and bacterial pneumonia [6]. Bacterial pneumonia is the most prevalent complication of influenza, occurring in about one in three patients who develop severe influenza. The microorganism most frequently isolated in patients with complicated influenza is *Streptococcus pneumonia* [7]. Bacterial pneumonia carries a heavy burden of morbidity: it is responsible for about 80% of hospital admissions involving patients with complicated influenza [6]. Two retrospective studies conducted in northeastern Italy from the years 2004 to 2013 on all pneumonia-related hospitalizations identified a mean annual rate ranging from 204.6 to 256.3 per 100,000 population, peaking among people over 65 (844.9 per 100,000 in males, 605.7 per 100,000 in females) [8,9].

Influenza can affect all age groups, but may cause severe disease or complications in higher-risk groups, such as the elderly, or people with underlying chronic conditions, although anyone may develop serious and even fatal complications [1]. In particular, the estimated number of deaths due to respiratory disease associated with influenza varies from 4.0 to 8.8 per 100,000 population every season, with the highest excess mortality among people over 75 years old [10].

Influenza and its complications are a major public health concern, and vaccination programs remain the most important effective strategy for reducing the number of cases. The World Health Organization (WHO) and the Council of the European Union recommend a vaccine coverage of at least 75% among individuals at greater risk of influenza-related complications [11,12].

Italy’s national influenza vaccination program recommends vaccination against influenza, and provides it free of charge to high-risk population groups, such as the elderly [13].

The primary goal of vaccination is to prevent the more severe forms of flu, particularly in the categories at highest risk of complications. The currently-used seasonal influenza vaccines contain the H1N1 strain, the H3N2 strain, and one or two B strains, i.e., they are trivalent or quadrivalent inactivated influenza vaccines, respectively. To increase their breadth of immunity, influenza vaccines have been produced in different formulations (subunit, split, virosomal, adjuvanted with MF59, and high-dose vaccine) and for different routes of administration (intranasal live-attenuated influenza vaccines or intradermic vaccines) [14]. In general, the evidence suggests that the currently-available influenza vaccine formulations are safe and well-tolerated, but there is interest in the development of universal influenza vaccines, because of the variability in their effectiveness from one season to another [15].

It is hard to demonstrate the efficacy of this preventive measure, which also depends on the mismatch that can occur between the circulating viral strains and the antigens included in the vaccine in the different flu seasons [16]. Few data are available on this issue as yet. A recent study conducted in Lazio (central Italy) in the 2016–2017 season found that vaccination reduced the risk of hospitalization and flu-related deaths [17].

The aim of the present study was to assess the impact of different influenza vaccination strategies in preventing hospital admissions for flu-related and other forms of pneumonia involving the elderly in Friuli Venezia Giulia (northeast Italy) during six consecutive influenza seasons from 2011–2012 to 2016–2017. Three different strategies were compared: an almost exclusive use of conventional vaccines (trivalent and quadrivalent inactivated vaccines); an almost exclusive use of enhanced vaccines, i.e., MF59-adjuvanted, intradermal, and virosomal; and a mixed use of one or the other type of vaccine. In a subsequent step, the analysis was restricted to currently-usable vaccines, and a vaccination strategy that envisages the almost exclusive use of conventional vaccines was compared directly with a strategy favoring the almost exclusive use of the enhanced MF59-adjuvanted trivalent inactivated vaccine (MF59-TIV).

## 2. Material and Methods

### 2.1. Design, Setting and Data Sources

We conducted a retrospective cohort study on data routinely collected by the health services in Friuli Venezia Giulia (FVG) in northeastern Italy, during six consecutive flu seasons: 2011/2012, 2012/2013, 2013/2014, 2014/2015, 2015/2016, and 2016/2017. At the time of the study, FVG had a population of about 1.2 million, with an average age of 46.2 years. Individuals aged 65 years or more accounted for 25.7% of the population (313,779 inhabitants). The FVG Regional Authority operates an automated, centralized system for recording and pooling health care data, funded by the Italian National Health Service, and patients are identified in the database by means of unique anonymous personal codes. For the purposes of this study, we considered the hospital discharge records, coded according to the International Classification of Diseases, Ninth Revision, Clinical Modification (ICD-9-CM), of all public and accredited private hospitals in the region, as well as data in the FVG vaccination registries.

### 2.2. Study Population and Influenza Vaccination Grouping Criteria

The study population included all people vaccinated for influenza at the age of 65 years or more and residing in one of the region’s five local health districts. In FVG, vaccination against the flu is recommended to everyone aged 65 years or more, regardless of their state of health, and it is offered free of charge [18]. During the study period, different influenza vaccines were available and used. Trivalent inactivated vaccines (TIVs) and quadrivalent inactivated vaccines were grouped together and classified as conventional vaccines (ConVs group), while MF59-TIV, intradermal TIV (ID-TIV), and virosomal TIV (VR-TIV) were grouped together and classified as enhanced vaccines (EnhVs group). Since ID-TIV and VR-TIV are not currently available for Italians aged 65 years and over, the second part of our study only considered MF59-TIV in the comparison with conventional vaccines.

To minimize any selection bias due to the use of enhanced vaccines for frail individuals, the percentage of use of each vaccine was calculated for each health district and each influenza season, and this percentage was used as a criterion for grouping people according to three different vaccination strategies, classifying the subjects in each group as follows: those belonging to the first group (ConVs Group) were managed according to a strategy that provides for the almost exclusive use of conventional vaccines (in at least 90% of cases); the second group (EnhVs group) included people covered by a strategy relying almost exclusively on enhanced vaccines (in 90% of cases or more); and individuals managed according to a strategy that provides for the use of both types of vaccine, each in less than 90% of cases, formed the third (MixVs group).

### 2.3. Study and Follow-up Periods

The six flu seasons were identified by the Italian Ministry of Health and were 16 January 2012 to 4 March 2012, 7 January 2013 to 24 March 2013, 20 January 2014 to 16 March 2014, 5 January 2015 to 15 March 2015, 25 January 2016 to 27 March 2016, and 19 December 2016 to March 5 2017. Allowing for immunological response after vaccination, the observation period started two weeks after vaccination, and in the absence of any event, ended with the end of the flu season, as listed above; in the event of pneumonia prompting hospitalization or death, the end of follow-up coincided with the date of the event concerned.

### 2.4. Outcomes

The study outcome was any hospitalization for which the patient’s discharge records indicated a respiratory condition as the principal diagnosis, but only for admissions within the flu season for which the individual had been vaccinated. The specific ICD codes considered were 481 for pneumococcal pneumonia (*Streptococcus pneumoniae* pneumonia) 482.9 for bacterial pneumonia, unspecified; 485 for bronchopneumonia, unspecified organism; 486 for pneumonia, unspecified organism; and 487 for influenza.

### 2.5. Statistical Analysis

A descriptive analysis was performed to characterize individuals’ demographics and clinical data. Categorical variables are reported as absolute numbers and percentages, and compared using the chi-square test or Fisher’s exact test. Continuous data are presented as means ± standard deviations (SD), and compared using Student’s *t*-test for unpaired data, performing a priori tests for the equality of variances. Trends over the years considered were assessed as average annual percent changes (AAPC).

Linear regression models were used to compare the hospital admission rate for pneumonia observed within different percentage levels of EnhV use, both by health district and flu season. Cox’s regression models were fitted to estimate the adjusted odds ratio (adj OR) of developing pneumonia requiring hospital admission. Models were adjusted for sex, age, vaccination against influenza and pneumonia, vaccination group, and seasonal mismatch, if any.

A *p*-value of <0.01 was accepted as statistically significant. The analyses were performed using the Statistical Package for the Social Sciences (SPSS 25.0; SPSS Inc., Chicago, IL, USA).

### 2.6. Ethics Statement

Complying with Italian legislation, all data used in this study were kept fully confidential. Patient identifiers had been substituted with anonymous codes before the database was accessed, so there was no way to identify the individuals concerned. Patients’ informed consent was not needed due to the anonymous nature of the routinely-collected data considered here. In Italy, such anonymized data may be analyzed in aggregate form for scientific purposes without further authorization [19]. The research also meets the requirements of the Declaration of Helsinki and, because it was an observational study, it was reported to the Padua Provincial Authority’s ethical committee.

## 3. Results

During the flu seasons considered (from 2011/2012 to 2016/2017), we identified a total of 987,266 individuals vaccinated against the seasonal flu virus, who were grouped as follows: 383,286 in the ConVs group (38.8% of the sample); 443,126 in the EnhVs group (44.9%); and 160,854 in the MixVs group (16.3%). Table 1 shows the characteristics of the sample by vaccination group. An overall 42.9% of the sample were male (423,151 individuals), with no significant differences in sex distribution between the groups. The mean age of the sample was 78.0 ± 7.7 years, and was also evenly distributed in the three groups.

**Figure 1:**
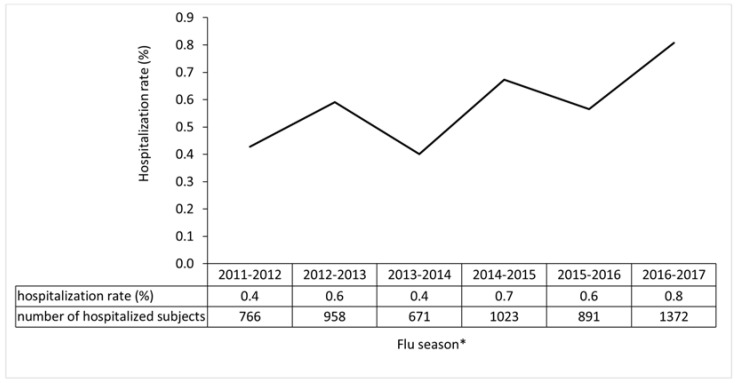
Hospitalization rate (%) and number of subjects with pneumonia by flu season. When the hospitalization rate was considered by vaccination group, the enhanced vaccines (EnhVs) group (2001 cases; 0.45%) and the conventional/enhanced vaccines (MixVs) group (1429 cases; 0.59%) had significantly lower rates than the conventional vaccines (ConVs) group (2251 cases; 0.75%), with *p* < 0.001. The difference in the number of admissions between the EnhVs Group and the MixVs Group was also significant (0.45% vs. 0.59%; *p* < 0.001). * AAPC = 10.8; 95% CI: −3.5–27.1.

There were an overall 5681 hospital admissions for pneumonia during the study period, i.e., 0.6% of the sample as a whole. The hospitalization rate tended to increase over the years, though not significantly (AAPC = 10.8 (95% CI: −3.5–27.1)), peaking in the 2016–2017 flu season with a hospitalization rate of 0.8% among all vaccinated individuals (Figure 1).

**Figure 2:**
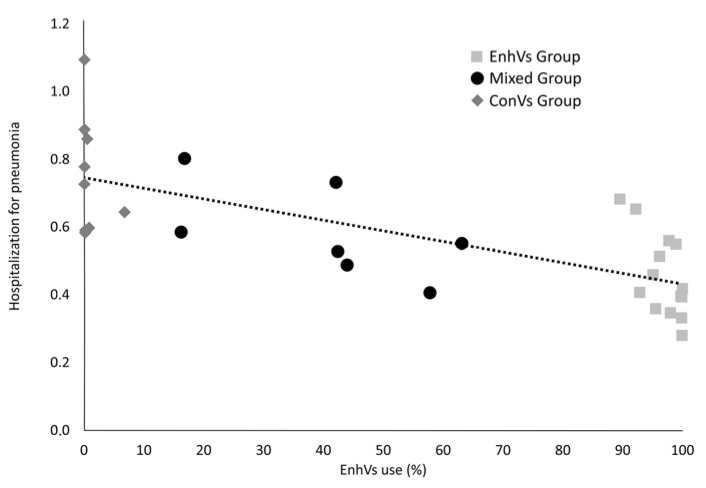
Relationship between level of enhanced vaccine use (%) and hospital admission rate (%) for pneumonia by health district and flu season. The line represents the linear regression equation. The correlation coefficient (*R*) was 0.7235 with a *p* < 0.001.

Pooling data for all flu seasons and health districts, an inverse linear relationship emerged, showing that the hospital admission rate for pneumonia as a percentage was inversely correlated with the percentage level of enhanced vaccine use (*R*^2^ = 0.5234; *p* < 0.001 by weighted linear regression) (Figure 2).

Multivariate analysis confirmed the significantly greater protective effect of both MixVs and EnhVs over a conventional vaccine strategy (adj OR = 0.81 (95% CI: 0.76–0.87) and 0.62 (95% CI: 0.59–0.66), respectively). It also emerged that having been vaccinated against the flu in previous years also has a protective effect (adj OR = 0.752 (95% CI: 0.695–0.814)). The analysis confirmed a direct association between older age, male sex, and H3N2 mismatch with a higher risk of hospitalization for pneumonia (Table 2).

Narrowing down the comparison to consider the vaccination strategies currently available for Italian adults aged 65 years or above, i.e., conventional versus enhanced vaccines, Table 3 shows the characteristics of the two subsamples. Out of a total of 479,397 individuals identified, 85.7% (*n* = 410,737) belonged to the ConVs Group, and 14.3% (*n* = 68,660) had been vaccinated with MF59-TIV. Overall, 42.7% of these individuals were male (*n* = 204,738), with no significant differences in their distribution between the two subsamples; and the average age was 78.1 ± 7.7 years. An overall 51.7% of these individuals had previously been vaccinated against *Streptococcus pneumoniae*. With a total of 3176 hospital admissions for pneumonia (0.66% of these individuals as a whole), the hospitalization rate was 0.48% in the MF59-TIV subsample, and 0.69% in the ConVs Group.

**Figure 3:**
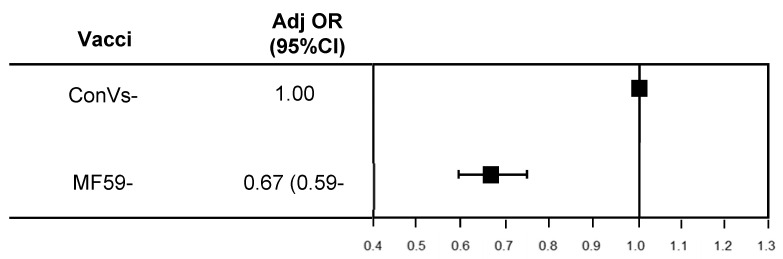
MF59-adjuvanted trivalent inactivated vaccine (MF59-TIV) vs. ConVs in preventing hospital admissions for pneumonia.

Comparing the MF59-TIV subsample with the conventionally vaccinated group in terms of hospital admissions for pneumonia, the results show that the former lowered the risk of hospitalization by 33% (adj OR = 0.67 (95% CI: 0.59–0.75); *p* < 0.001) (Figure 3).

Multivariate analysis confirmed that age (adj OR = 1.103 (95% CI: 1.098–1.108)), male sex (adj OR = 1.859 (95% CI: 1.730–1.997)), H3N2 mismatch (adj OR = 1.359 (95% CI: 1.253–1.473)), were associated with the risk of hospitalization for pneumonia, and that previous vaccination against the flu in previous years also has a protective effect (adj OR = 0.744 (95% CI: 0.674–0.821))

## 4. Discussion

Worldwide, infections of the lower respiratory tract are one of the main causes of morbidity and mortality, especially among the elderly, and there is evidence of a rising trend of their frequency in many regions [20], a picture also confirmed by our data. This study aimed to ascertain the importance of the influenza vaccination strategy adopted for elderly people, with a view to reducing hospital admissions for flu-related pneumonia. Vaccination against seasonal flu is an important preventive measure in this age group, but its efficacy varies, depending on the vaccine coverage rate, the type of strain circulating, any mismatch between the circulating strain and the strain included in the vaccine’s formulation, the type of vaccine used, and the vaccination strategy adopted [21].

For now, our study seems to be one of the few to have assessed the risk of hospitalization for respiratory disease in relation to different influenza vaccination strategies. The strength of the study lies in the large size of the sample considered and the long period of observation, which enabled us to analyze different strategies and different percentages of circulating virus strains. Although we lacked information on hospitalized patients’ specific underlying diseases, our analysis of different vaccination strategies in the population as a whole will have minimized this bias. Based on a regional directive, the enhanced vaccine was probably administered to individuals in the MixVs group, who had more underlying diseases and were living in long-term facilities. Nonetheless, our results should be interpreted with caution, because this study has several limitations. First, the “efficacy of vaccination” outcome was not confirmed, although the ICD diagnostic codes identifying patients with pulmonary disease hospitalized during the flu season probably concern cases of influenza-related diseases. Second, the lack of an unvaccinated cohort (since it was impossible to glean from our database of vaccination records) prevented us from comparing our findings with a control group. That said, published data indicate that annual influenza vaccination reduced the risk of influenza-related hospital admissions when vaccinated and unvaccinated subjects were compared [22].

In our study, the mean age of patients at the time of their hospital admission for pneumonia was 83.5 ± 7.8 years, which is consistent with recent findings [23] showing a direct correlation between pneumonia hospitalization and age. A weakening immune system, combined with age-related physiological changes occurring in the airways, can make the elderly more vulnerable to respiratory infections, thus explaining the increasing burden of pneumonia with aging [24]. It is less easy to explain the higher frequency of hospitalizations involving the male sex seen in our study (0.7% vs. 0.5% in females). In Europe as a whole, the rate of comorbidity is higher among women, and the difference is accentuated with aging, but this difference has not been confirmed in Italy [25]. Other studies have revealed differences in the type of comorbidity by sex, with men having a higher prevalence of cancer, ischemic heart disease, and kidney failure than women [26], which might explain the former’s greater vulnerability to respiratory infectious diseases.

From the results of our analysis, it emerged that the risk of hospitalization for respiratory diseases positively correlated with H3N2 mismatch (adj OR = 1.440 (95% CI = 1.347–1.540) in the sample as a whole, and adj OR = 1.398 (95% CI 1.291–1.514) when the analysis was restricted to individuals vaccinated with conventional vaccines or MF59-TIV). Compared with other strains, the influenza A (H3N2) virus is more frequently liable to natural antigenic changes, giving rise to a mismatch between strains contained in current flu vaccines and circulating strains. Our finding concerning the influence of such a mismatch is consistent with other reports on elderly people [27,28]. In the 2016/2017 flu season, the H3N2 strain accounted for about 90% of the positive specimens stockpiled for virological surveillance purposes [17], and it was in this same flu season that a study conducted in some areas showed a lack of vaccine efficacy in preventing emergency department visits, hospitalizations, or deaths due to influenza or pneumonia [29]. This would point to the importance of using a more effective vaccination strategy, such as enhanced vaccines, in the elderly.

A possible decline in the protection conferred by the vaccine might also be due to mutations that occur during the vaccine production process in eggs (egg-adapted mutation), which particularly affect the H3N2 strain [30]. This would also suggest giving priority to the production and use of more effective, improved seasonal vaccines, such as adjuvanted-cell-based vaccines and higher vaccine doses [31,32].

We found an inverse linear relationship between hospital admissions for pneumonia and the percentages of enhanced vaccine (correlation coefficient *R* = 0.7235; *p* < 0.001)—i.e., MF59-TIV, virosomal TIV, and intradermal TIV—used each season. Several studies have shown that these enhanced vaccines are more immunogenic, and more effective than inactivated vaccines in preventing hospitalization for influenza and pneumonia [21,33,34,35].

Finally, our study supports the results of a recent work conducted in Italy showing that MF59-TIV seems to reduce the risk of hospitalization for pneumonia by about 40% compared with non-adjuvanted flu vaccines [36]. In fact, we found this risk to be 33% lower when MF59-TIV was used extensively.

Adjuvanted influenza vaccines induce a greater and broader immune responses in elderly people with underlying chronic conditions (especially the H3N2 strains), also potentially affording clinical benefits in seasons when an antigenic mismatch occurs [37,38].

A recent meta-analysis has suggested that adjuvanted MF59 vaccine is effective in reducing several influenza-related outcomes among the elderly. MF59-TIV was found to be highly effective (94% (95% CI: 47–100%)) in reducing influenza-like illness among institutionalized elderly. MF59-TIV also displayed a greater efficacy than non-adjuvanted vaccines in preventing hospitalizations due to pneumonia/influenza (adjusted risk ratio = 0.75 (95% CI: 0.57–0.98)) and laboratory-confirmed influenza (adjusted odds ratio = 0.37 (95% CI: 0.14–0.96)) [32].

## 5. Conclusions

The present study contributes to the evidence indicating that enhanced influenza vaccines, and MF59-adjuvanted vaccines in particular, are more effective in the elderly than conventional vaccination strategies. It also confirms that H3N2 flu seasons coincide with a greater burden of disease in older people.

## Figures and Tables

**Table 1:** Individuals vaccinated against the flu in the Friuli Venezia Giulia region by vaccination strategy and characteristics, in six consecutive flu seasons (2011/2012 to 2016/2017).

Characteristics	Total		Vaccination Strategy					
			ConVs		MixVs		EnhVs	
	(*n* = 987,266)		(*n* = 383,286)		(*n* = 160,854)		(*n* = 443,126)	
Sex (*n* (%))								
Male	423,151	(42.9)	128,740	(42.6)	104,256	(43.1)	190,155	(42.9)
Female	564,115	(57.1)	173,325	(57.4)	137,819	(56.9)	252,971	(57.1)
Age group (*n* (%))								
65–74 years old	389,943	(39.5)	113,661	(37.6)	96,083	(39.7)	180,199	(40.7)
75–84 years old	396,511	(40.2)	125,337	(41.5)	97,067	(40.1)	174,107	(39.3)
85+ years old	200,812	(20.3)	63,067	(20.9)	48,925	(20.2)	88,820	(20.0)
Days of follow-up (mean (SD))	118.2	(15.3)	116.7	(16,1)	118.8	(14.6)	119.0	(15.0)
Prior pneumonia vaccination	529,004	(53.6)	194,447	(50.7)	85,361	(53.1)	249,196	(56.2)
Hospitalization for pneumonia	5681	(0.58)	2251	(0.75)	1429	(0.59)	2001	(0.45)

*n*: Number of subjects.

**Table 2:** Distribution of the characteristics of the sample by need for hospital admission for pneumonia.

Variables	Hospitalization		OR (95% CI)	Adj OR (95% CI)
	Yes	No		
Age (means ± SD)	83.5 ± 7.8	77.9 ± 7.7	**1.091 (1.087–1.094)**	**1.099 (1.096–1.103)**
Sex [*n* (%)]				
Female	2785 (0.5)	561,330 (99.5)	Ref.	Ref.
Male	2896 (0.7)	420,255 (99.3)	**1.389 (1.312–1.463)**	**1.822 (1.727–1.922)**
Vaccination group [*n* (%)]				
ConVs group	2819 (0.7)	380,467 (99.3)	Ref.	Ref.
MixVs group	861 (0.5)	159,993 (99.5)	**0.726 (0.672–0.784)**	**0.813 (0.760–0.869)**
EnhVs group	2001 (0.5)	441,125 (99.5	**0.612 (0.578–0.648)**	**0.622 (0.58–0.661)**
H3N2 mismatch [*n* (%)]				
no	3286 (0.5)	662,288 (99.5)	Ref.	Ref.
yes	2395 (0.7)	319,297 (99.3)	**1.512 (1.434–1.594)**	**1.471 (1.379–1.570)**
B mismatch [*n* (%)]				
No	1023 (0.7)	150,964 (99.3)	Ref.	Ref.
Yes	4658 (0.6)	830,621(99.4)	1.207 (1.128–1.291)	1.041 (0.958–1.131)
Previous vaccination against influenza [*n* (%)]				
No	733 (0.5)	136,373 (99.5)	Ref	Ref.
Yes	4948 (0.6)	845,012 (99.4)	**0.917 (0.842–0.992)**	**0.752 (0.695–0.814)**
Pneumococcal vaccination (*n* (%))				
No	2517 (0.5)	455,743 (99.5)	Ref.	Ref.
Yes	3164 (0.6)	525,807 (99.4)	**1.090 (1.034–1.148)**	1.032 (0.979–1.089)

Statistically significant data in bold. *n*: number of subjects.

**Table 3:** Characteristics of individuals in the Friuli Venezia Giulia region vaccinated with either a conventional vaccine or MF59-TIV in six consecutive flu seasons (2011/2012–2016/2017).

Characteristics	Total		Type of Vaccination			
			ConVs Group		MF59-TIV	
	(*n* = 479,397)		(*n* = 410,737)		(*n* = 68,660)	
Sex (*n* (%))						
Male	204,738	(42.7)	176,154	(42.9)	28,584	(41.6)
Female	274,659	(57.3)	234,583	(57.1)	40,076	(58.4)
Age group (*n* (%))						
65–74 years old	162,486	(33.9)	139,914	(34.1)	22,572	(32.9)
75–84 years old	202,083	(42.2)	174,475	(42.5)	27,608	(40.2)
85+ years old	114,828	(24.0)	96,348	(23.5)	18,480	(26.9)
Days of follow-up (mean (SD))	117.1	(15.9)	116.8	(16.1)	118.7	(14.8)
Prior vaccination for pneumonia	247,939	(51.7)	204,188	(49.7)	43,751	(63.7)
Hospitalization for pneumonia	3176	(0.66)	2849	(0.69)	327	(0.48)

*n*: number of subjects.
